# The Analog-to-Digital Evolution of Neurosurgery: Ethics and Professionalism from Scalpels to Robots

**DOI:** 10.3390/neurosci7030065

**Published:** 2026-06-04

**Authors:** Petar Vuleković, Mario Ganau, Lukas Rasulić, Đula Đilvesi, Jagoš Golubović

**Affiliations:** 1 Faculty of Medicine, University of Novi Sad, Hajduk Veljkova 3, 21000 Novi Sad, Serbia; djula.djilvesi@mf.uns.ac.rs (Đ.Đ.); jagos.golubovic@mf.uns.ac.rs (J.G.); 2 Department of Neurosurgery, University Clinical Centre of Vojvodina, Hajduk Veljkova 1, 21000 Novi Sad, Serbia; 3 Nuffield Department of Clinical Neurosciences, University of Oxford, Oxford OX3 9DU, UK; mario.ganau@alumni.harvard.edu; 4 Department of Neurosurgery, Oxford University Hospitals NHS Foundation Trust, Oxford OX3 9DU, UK; 5 School of Medicine, BAU International University Batumi, 237 Fridon Khalvashi ST, 6010 Batumi, Georgia; 6 Faculty of Medicine, University of Belgrade, Dr Subotića Starijeg 8, 11000 Belgrade, Serbia; lukas.rasulic@gmail.com; 7 Department for Peripheral Nerve Surgery, Functional Neurosurgery and Pain Management Surgery Clinic for Neurosurgery, Clinical Center of Serbia, Dr Koste Todorovića 4, 11000 Belgrade, Serbia

**Keywords:** digital neurosurgery, narrative review, ethics, professionalism, neuronavigation, brain shift, eloquent cortex, stereotactic robotics, intraoperative neurophysiology, skull base surgery

## Abstract

Introduction: Neurosurgery has evolved from an anatomy-driven analog discipline into a digitally augmented field supported by multimodal imaging, neuronavigation, intraoperative imaging, neurophysiological monitoring, robotics, augmented reality, and artificial intelligence. Objective: To examine how this transition has altered professional responsibility, informed consent, training, and medico-legal accountability in neurosurgical practice. Methods: We performed a structured narrative review of the literature on digital neurosurgery and its ethical and professional implications, focusing on publications from 1990 onward and supplemented by landmark historical papers. Sources were selected for relevance to cranial, spinal, skull base, stereotactic, and neuro-oncological neurosurgery, and then synthesized into thematic domains including brain shift, eloquent cortex preservation, stereotactic accuracy, intraoperative neurophysiology, workflow integration, equity, and liability. Results: Digital systems improve lesion localization, function-preserving surgery, stereotactic precision, documentation, and training, but they also introduce new vulnerabilities related to registration error, brain shift, platform dependence, data overload, cost, cybersecurity, deskilling, and diffuse accountability. Conclusions: Digital augmentation expands rather than diminishes the neurosurgeon’s responsibility. The neurosurgeon remains accountable for surgical indication, interpretation of technology-generated information, intraoperative override, and communication of technology-specific risks. The central ethical challenge is to integrate digital tools without weakening patient-centered judgment.

## 1. Introduction

Not long ago, a neurosurgeon planning a complex brain operation relied primarily on hand-drawn sketches and two-dimensional X-ray films displayed on a lightbox. Orientation in the operating room depended on the surgeon’s intimate knowledge of anatomy and occasionally a simple mechanical frame to guide an instrument’s trajectory. Today, by contrast, neurosurgeons work in digital operating rooms equipped with high-definition screens, real-time neuronavigation systems that register instruments to patient-specific imaging datasets, and robotic assistants capable of sub-millimeter accuracy. The transformation from these analog beginnings to the current digital era represents one of the most significant paradigm shifts in the history of neurosurgery [1,2].

This review examines the ethical and professional implications of the analog-to-digital transition in neurosurgery through a literature-based narrative synthesis informed, but not driven, by intergenerational surgical experience. The perspectives of authors trained in pre-digital and digitally mature eras are retained as an interpretive lens for understanding how responsibility, consent, technical judgment, and training have changed over time.

The review has three aims. First, it summarizes the major technological transitions that reshaped modern neurosurgical practice. Second, it identifies the neurosurgery-specific domains in which digital systems most directly alter decision-making, including navigation fidelity in deformable brain tissue, preservation of eloquent cortex and white matter tracts, stereotactic precision, skull base workflow integration, and function-preserving surgery. Third, it analyzes how these technologies redistribute, but do not eliminate, professional responsibility, with particular attention to informed consent, documentation, accountability, and equity of access.

The sections that follow therefore move from historical evolution to structured thematic synthesis. A shortened illustrative thought experiment is included only to contextualize ethical contrasts between eras and is not presented as clinical evidence.

## 2. Review Methodology

This study was designed as a structured narrative review of the analog-to-digital transformation of neurosurgery, with emphasis on its ethical, professional, and medico-legal implications. The narrative format was selected because the topic spans heterogeneous historical, technical, educational, ethical, and legal literature that is not suitable for quantitative pooling, but still requires transparent sourcing and structured thematic synthesis.

A literature search was conducted in PubMed/MEDLINE, Scopus, and Web of Science using combinations of terms related to neurosurgery, neuronavigation, robotics, augmented reality, virtual reality, artificial intelligence, brain shift, eloquent cortex, awake mapping, intraoperative neurophysiology, stereotactic surgery, skull base surgery, ethics, professionalism, informed consent, training, and liability. English-language publications from 1990 onward were prioritized and supplemented with landmark historical reports relevant to the pre-digital era. Reference lists of key papers were also screened to identify additional relevant sources.

Sources were considered eligible if they addressed the clinical use, historical development, ethical implications, professional consequences, or medico-legal dimensions of digital technologies in neurosurgery. We prioritized publications directly relevant to cranial, spinal, skull base, neuro-oncological, functional, and stereotactic neurosurgery. General surgical literature was included only when it provided a conceptual, ethical, or regulatory framework directly applicable to neurosurgical practice. Publications focused exclusively on engineering performance without clinical or ethical relevance were not emphasized in the synthesis.

The selected literature was synthesized thematically rather than statistically. The final narrative was organized around the following domains: the analog foundations of neurosurgical practice; digital transformation in contemporary neurosurgery; brain shift and navigation fidelity; preservation of eloquent cortex and white matter tracts; stereotactic accuracy and robotics; skull base and multimodal workflow integration; training and skill retention; equity, access, and cost; and professional accountability in technology-mediated care.

## 3. Literature Synthesis and Discussion

### 3.1. The Analog Era: Foundations and Limitations

Modern neurosurgery builds upon pioneers who practiced in an analog environment, where tactile skill, direct vision, and rudimentary imaging defined clinical practice. Surgeons such as Harvey Cushing relied on surface anatomy and landmark-based knowledge, aided by basic X-rays, ventriculography, or myelography to localize lesions indirectly. Stereotactic frames (e.g., Leksell, BRW) and operating microscopes allowed precise targeting and visualization, yet planning depended on manual calculation and mental reconstruction of two-dimensional data [3,4,5,6].

While effective in skilled hands, the analog era imposed clear limitations. Imaging was low-resolution and static, intraoperative navigation relied on memory, and information sharing was slow. Surgeons compensated through extensive training, personal judgment, and ingenuity, which fostered a culture of self-reliance but also highlighted the need for technological innovation. Ethically and professionally, responsibility was concentrated more directly on the operating neurosurgeon because decision support was limited and device mediation minimal, although institutional and material constraints still shaped what could be offered safely: there were no algorithmic aids or automated systems to mediate decision-making.

### 3.2. The Digital Revolution in Neurosurgery

The digital transformation of neurosurgery is not defined merely by improved visualization; it is defined by the integration of multimodal data into operative judgment. In neuro-oncology, neuronavigation, tractography, fluorescence guidance, intraoperative ultrasound, and intraoperative MRI support lesion localization, corridor design, and assessment of extent of resection. In surgery near eloquent cortex, functional MRI, navigated transcranial magnetic stimulation, awake mapping, and intraoperative neurophysiology help the surgeon balance cytoreduction against preservation of motor, language, and cognitive function [7,8,9,10,11,12]. In stereotactic practice, navigation and robotics influence the safety and precision of biopsy, deep brain stimulation, and stereoelectroencephalography trajectories, where millimetric error can change both efficacy and risk. In skull base and deep-corridor surgery, endoscopy, exoscopy, augmented overlays, and team-wide digital visualization alter not only what the surgeon sees, but how the entire workflow is coordinated [13,14,15,16,17].

For neurosurgery, therefore, digital innovation changes more than convenience. It changes how anatomical truth is generated, updated, verified, and acted upon in an organ that is deformable, functionally eloquent, and intolerant of small technical errors.

The advent of CT and MRI in the late 20th century introduced high-resolution, non-invasive visualization, forming the basis for computer-assisted surgery. Frameless neuronavigation allowed real-time tracking of instruments relative to patient anatomy, akin to a GPS system, while robotic systems gradually enhanced precision and reduced fatigue in both cranial and spinal procedures [7,8,9,10,11,12]. Advanced visualization platforms, exoscopes, and augmented reality (AR) now provide the entire surgical team with real-time imaging overlays, improving communication, safety, and teaching [13].

Minimally invasive approaches, supported by navigation, robotics, and intraoperative imaging, have transformed patient care—reducing tissue trauma, hospital stays, and recovery time [14,15,16]. Intraoperative fluorescence, tractography, and real-time imaging further enhance precision [17].

### 3.3. Neurosurgery-Specific Ethical and Professional Domains

Brain shift and navigation fidelity. Unlike many other surgical fields, neurosurgery often operates in deformable tissue within a rigid cranial frame. Once cerebrospinal fluid is released, the dura is opened, the patient is positioned, or debulking begins, preoperative image registration may progressively lose fidelity. Ethical use of neuronavigation therefore requires not merely access to the technology, but judgment about when its assumptions are no longer reliable and when direct anatomy, intraoperative ultrasound, intraoperative MRI, or functional mapping must take precedence.

Eloquent cortex and white-matter preservation. In glioma and epilepsy surgery, the ethically relevant endpoint is not resection alone but resection consistent with preservation of meaningful function. Functional MRI, tractography, awake mapping, motor evoked potentials, and related modalities reduce uncertainty but do not eliminate trade-offs between oncological ambition and neurological risk. For that reason, technology does not neutralize value-laden decisions; it makes them more data-rich while leaving the final responsibility with the neurosurgeon.

Stereotactic accuracy and robotics. In biopsy, deep brain stimulation, and stereoelectroencephalography, the ethical stakes of precision are unusually high because millimetric deviations may affect hemorrhage risk, diagnostic yield, target engagement, and postoperative function. Robotic systems may enhance reproducibility, but they also increase dependence on registration accuracy, trajectory planning, hardware integrity, and software verification. Accountability therefore includes verifying the digital plan rather than merely executing it.

Skull base and multimodal workflow integration. In skull base and deep-seated cranial surgery, digital tools alter not only dissection but team cognition. Endoscopy, exoscopy, augmented overlays, and navigation can improve shared orientation in narrow or deep operative corridors, yet they can also introduce display clutter, latency, and overconfidence in virtual anatomy. In this setting, professional responsibility includes orchestration of the digital workflow as well as technical performance.

Across these domains, the central ethical shift is that responsibility becomes technologically mediated but not technologically displaced. The neurosurgeon remains responsible for indication, interpretation, verification, override, and communication of digital risk [18,19]. A side-by-side comparison of analog- and digital-era neurosurgery across diagnostic, operative, ethical, and infrastructural domains is summarized in Table 1.

### 3.4. Comparison and Ethical Reflection

Both eras uphold core ethical principles—beneficence, autonomy, and professional integrity—within their respective technological contexts. The future challenge is ensuring technology enhances human-centered care without supplanting clinical judgment. Yet, this technological edge brings new ethical and legal dimensions:

- Consent and understanding: in awake craniotomies for eloquent-area gliomas, in deep brain stimulation for movement disorders, and in robot-assisted spinal pedicle screw placement, patients must be helped to grasp how AI-assisted planning, intraoperative imaging, and machine-guided trajectories shape both the proposed benefit and the residual margin of error [20,21,22].
- Accountability: when a neuronavigation system misregisters because of brain shift, when an AR overlay misaligns over an arteriovenous malformation, or when a robotic platform places a screw outside the planned corridor, the operating neurosurgeon—not the software vendor or the device—retains legal and professional responsibility for the intraoperative decision [23,24,25].
- Equity: intraoperative MRI, 5-ALA fluorescence, exoscopic systems, and robotic spinal platforms remain concentrated in well-resourced centers, so a patient with a glioma or a complex spinal deformity may receive markedly different standards of care depending on geography rather than on disease biology [26,27,28].
- Human connection: the neurosurgical consultation—discussing a newly diagnosed glioblastoma, a ruptured aneurysm, or a pediatric posterior fossa tumor with the family—must not be displaced by screens, dashboards, and automated risk scores; precision tools should support, not substitute for, the bedside conversation that defines neurosurgical care [29,30,31].

The challenge for future neurosurgeons will not be choosing between analog and digital medicine, but ensuring that technology serves humanity, not the other way around; the goal of neurosurgery today is to maximize the advantages of digital tools while minimizing their downsides.

### 3.5. Benefits, Risks, and Mitigation Strategies

Digital technologies are most valuable in neurosurgery when they solve characteristically neurosurgical problems: localization in deformable tissue, preservation of eloquent function, and millimetric stereotactic targeting. Their advantages therefore include improved spatial planning, better documentation of extent of resection, enhanced team visualization, expanded simulation-based training, and more precise integration of anatomical and functional information [32,33,34,35,36,37,38].

These gains are inseparable from new failure modes. Digital neurosurgery introduces risks related to registration error, brain shift, multimodal data overload, platform dependence, reduced fallback confidence in manual techniques, cybersecurity, widening inequity between centers, and uncertainty about distributed liability. The relevant professional question is therefore not whether digital tools should be accepted or rejected in principle, but how they can be integrated with verification steps, manual override capacity, structured consent, and context-aware documentation. The main domains, advantages, risks, and mitigation strategies are summarized in Table 2.

## 4. Conclusions

The analog-to-digital transformation of neurosurgery has improved visualization, targeting, function-preserving surgery, and documentation, but it has not diminished the neurosurgeon’s accountability. If anything, responsibility has broadened: the neurosurgeon must now indicate surgery appropriately, interpret multimodal data critically, recognize the limits of navigation and automation, communicate technology-specific risks, and document technology-mediated decisions with greater precision.

The key ethical questions arise where digital assistance is least straightforward—when the brain shifts, when eloquent networks are at risk, when millimetric stereotactic error matters, and when multiple digital systems influence intraoperative judgment. The value of digital neurosurgery should therefore be judged not by technological spectacle, but by whether it delivers safer, function-preserving, equitable, and professionally accountable care.

The unifying theme is integration of analog and digital skills, human judgment and technology, high-tech solutions and patient-centered care. Thoughtful application of training, design, policy, and ethics can mitigate the downsides of digital neurosurgery. Just as innovation drove the field forward, continued innovation is required to address the new challenges that accompany technological advancement. Ultimately, technology should enhance, not replace, the moral agency, clinical wisdom, and human responsibility of the neurosurgeon.

## Figures and Tables

**Table 1:** Comparison of Analog vs. Digital Neurosurgery: Case-Based and Conceptual Overview.

Domain	Analog Era	Digital Era	Key Ethical/Legal Considerations
Diagnosis & Imaging	CT-based localization; thick-slice MRI on film; histology only	3T MRI, fMRI, DTI, intra-op MRI; molecular profiling (IDH, MGMT); AI-assisted pathology	Informed consent must address advanced imaging, genetics, and AI data use
Preoperative Planning	Hand sketches; anatomical landmarks; frame-based stereotaxy	Neuronavigation; AR/VR rehearsal; 3D digital modeling	Data accuracy and documentation of digital workflow are legal safeguards
Intraoperative Guidance	Optical microscope; basic ultrasound; tactile feedback dominant	HD exoscope; AR overlay; real-time navigation; fluorescence (5-ALA)	Technology does not transfer liability—surgeon retains full accountability
Extent of Resection	Approximate subtotal resection; no real-time confirmation	Imaging-confirmed near-total resection; fluorescence-guided surgery	“Maximal safe resection” dynamically balanced with functional preservation
Adjuvant Treatment	Radiotherapy + BCNU; limited protocols	Stupp protocol; TTF; immunotherapy; molecular-driven trials	Equity concerns—access varies by region and income
Ethical Model	Paternalistic decision-making	Shared decision-making; patient autonomy	Digital visualization improves understanding but requires literacy and time
Standard of Care	Based on technical skill and experience	Evidence-based digital standards; documented navigation and imaging use	Legal definition evolves with technological adoption
Training & Skills	Apprenticeship; anatomy labs; high tactile reliance	Simulation, VR/AR, AI assessment; reduced tactile input	Must preserve core surgical judgment alongside digital proficiency
Infrastructure & Resources	Low-cost setup	High-cost technology (navigation, iMRI, AR)	Global justice challenge in access to modern neurosurgery
Outcomes	Subtotal resection typical; survival measured in months in high-grade glioma series	Higher near-total resection rates; survival gains reflect combined advances in tumor biology, adjuvant therapy, and patient selection	Cross-era comparisons confounded by molecular classification, adjuvant regimens, and case selection; outcomes cannot be attributed to digital tools alone

**Table 2:** Advantages, Limitations, and Mitigation Strategies in the Digital Era of Neurosurgery.

Domain	Advantages	Risks/Limitations	Mitigation Strategies
Precision & Safety	Real-time navigation, AR overlays, intra-op imaging, fluorescence → greater accuracy, lower morbidity	Device overreliance; calibration/registration errors	Mandatory system checks; redundant verification; surgeon cross-validation
Extent of Resection	Intraoperative MRI and fluorescence enable near-total removal	High cost; limited availability; workflow delays	Portable/scalable imaging; shared regional centers; outcome-based funding
Training & Skills	VR/AR simulation shortens learning curve; risk-free repetition	Potential loss of tactile/manual expertise	Hybrid curricula combining simulation with analog microsurgical training
Data & Decision Support	Integration of imaging, genomics, AI → personalized planning	Data overload; interpretive bias; cybersecurity risks	Curated dashboards; human-in-the-loop AI review; strong encryption/governance
Patient Engagement	3D visualization enhances understanding and consent quality	Digital literacy gaps; information overload	Simplified visuals; plain-language summaries; structured consent sessions
Ethical & Legal Framework	Transparent documentation; traceable decisions	Surgeons remain liable for AI/robotic errors; complex consent	Shared liability frameworks; manual override capability; detailed documentation
Access & Equity	Telemedicine and digital education expand expertise globally	Technology gap increases disparity between centers	Subsidized technology; open-source platforms; global training programs
Human Dimension	Improved team coordination through shared visualization	Risk of depersonalization	Preserve direct dialogue; embed empathy training in digital workflows
Economics	Fewer complications and shorter stays may reduce long-term costs	High upfront investment and maintenance burden	Value-based procurement; shared infrastructure; public–private partnerships
Research & Innovation	Continuous data capture accelerates trials and translational work	Data ownership and secondary-use concerns	Clear data-use agreements; anonymized research consent pathways

## Data Availability

No new data were created or analyzed in this study. Data sharing is not applicable to this article.

## Abbreviations

The following abbreviations are used in this manuscript:

AR	Augmented Reality
AI	Artificial Intelligence
MIS	Minimally Invasive Surgery
CT	Computed Tomography
MRI	Magnetic Resonance Imaging
VR	Virtual Reality
fMRI	Functional Magnetic Resonance Imaging
DTI	Diffusion Tensor Imaging
IDH	Isocitrate Dehydrogenase
MGMT	O6-Methylguanine-DNA Methyltransferase
HD	High Definition
5-ALA	5-Aminolevulinic Acid
BCNU	Carmustine (Bis-chloroethylnitrosourea)
TTF	Tumor Treating Fields
HGG	High-Grade Glioma
OR	Operating Room
HIFU	High-Intensity Focused Ultrasound
